# American Academy of Dental Science

**Published:** 1896-12

**Authors:** 

**Affiliations:** American Academy Dental Science


### AMERICAN ACADEMY OF DENTAL SCIENCE.

The regular monthly meeting of the American Academy of Dental Science was held at Young's Hotel, Boston, May 6, at six o'clock.

The society took up the discussion of subjects formulated by the American Dental Association.

*Dr. Fillebrown.*—Mr. President, my plan for the opening of the discussion of the questions proposed by the committee of the American Dental Association is to suggest to you an answer to each question, which, to my mind, would be suitable to send as the society's answer. They are simply suggestions, and the society can accept them or not, as it chooses. Perhaps this plan will not meet the approbation of the Academy; if not, some one else may offer a better one during the discussion.

The first question is—

"Should not the appointment of Dental Examining Boards be under the control of the State dental societies?"

In opening the discussion of this question, all I should say would be,—

Nominations for Dental Examining Boards should receive the endorsement of the profession of the State; ordinarily the State dental societies should nominate candidates for the board.

That seems to me to be a statement of facts, and I do not think that multiplying words will make it clearer; consequently I will await your discussion.

*Chairman Barker.*—Gentlemen, you hear the answer proposed by Dr. Fillebrown. What is your pleasure regarding it?

If there is to be no discussion of this question, I suppose it can be considered tantamount to the endorsement of the Academy of the position assumed by Dr. Fillebrown.

*Dr. Eames.*—The State dental societies usually having a larger representation than the other dental societies, it seems to me that, by this broad representation of the profession in the State, more dentists could have a share in the appointment of the Examining Board than as now arranged. Therefore I move you, Mr. President, that the answer, as formulated by Dr. Fillebrown be adopted.

So voted.

*Dr. Fillebrown.*—The next question, Mr. President, is—

"Should not the granting of certificates of qualifications by Examining Boards to non-graduates be generally abolished?"

I would suggest as an answer:

All dental laws should require a degree as a condition for examining and license.

I firmly believe that in whatever line a person is called upon to be examined, he should have his degree, specially qualifying him in that calling, and it seems to me that if a person wishes to enter upon the practice of dentistry he should have in the first place, his certificate of graduation from some reputable dental school. This would show that he has passed his studentship and a creditable examination before his teachers. I hope my answer will be endorsed.

*Dr. Werner.*—Does the law in this State prescribe that the Examining Board should have degrees themselves?

*Dr. Fillebrown.*—No, it does not even require that dental students should have degrees.

*Dr. Werner.*—If you require the applicants to have a degree, should you not require the members of the Examining Board to have degrees? I approve of Dr. Fillebrown's answer. I think applicants should be obliged to have a degree, and the Examining Board itself have qualifications other than political influence.

*Dr. Bradley.*—I am of the opinion that several undergraduates have passed the State examination in this State, and are practising, who failed to secure their degree from the dental school. They took their examinations before the State board just previous to the examinations at the school, and, though failing to graduate, nevertheless received their certificate of qualification from the board.

*Dr. Belyea.*—What harm is there in allowing a man to go up for examination without having a degree? I do not see why any man who thinks he is capable of practising dentistry should not be allowed to take the examination and prove that he is worthy of registration.

*Dr. Werner.*—I feel the whole spirit of Dr. Belyea's remarks; at the same time we must look deeper into the question. The examining boards are an established fact, and they have an immense power. The dental colleges are beginning to feel that their decisions as to who shall practise dentistry cannot always be approved, especially in the case of students, who, if they pass the Examining Boards, do not care for the degrees. I would like to see the Examining Boards differently appointed as well as being themselves obliged to possess degrees.

*Dr. Belyea.*—It seems to me the gentleman is talking on the first question, which has already been discussed and passed. We are not considering the appointment of the Examining Boards now; the question is, Shall we stop a man from taking the examination to see whether he is fitted for the practice of dentistry or not? It seems to me if you stop that you strike a blow at the very foundation of justice. I do not believe in it.

*Dr. Potter.*—I believe that Dr. Fillebrown's answer should be accepted as the answer of the Academy to this question. If the student can get his license before he has graduated from the dental school, he will say to himself, "If the State of Massachusetts says that I am a competent dentist, why should I pay attention to these men in the dental school who are trying to make me more competent?" The importance of subsequent instruction, in the mind of the man who has already secured his license to practise previous to his graduation, must thus be lessened.

*Dr. Allen.*—We have known of cases of students who have passed the Board of Examiners with something less than a year's experience in dentistry,—a fact which should speak for itself in this connection.

*Dr. Belyea.*—That is a matter which should be regulated from other sources; that is a point where you must attack the State Board and not the students who come up for examinations. Perhaps the student was well prepared for a harder examination. I do not think it is fair, nor in the line with our customary views of liberty and justice, to prevent a man from taking an examination to see if he is properly qualified to practise dentistry simply because he has not a degree.

*Dr. Williams.*—The whole question seems to me to hang on this balance, whether the Examining Boards and their queries are a more thorough, more rigid test of a man's qualifications than is the examination at the close of the dental college. If the Examining Board is better qualified to judge of a man's capabilities than college faculties, then I do not see why anybody should not have the right to go before them for examination; if, on the other hand, the college faculties are better qualified to say whether a man is sufficiently capable to practise dentistry or not, then it would, of course, be advisable that the candidate for examination before the board should be required to present a degree. In some States the appointment of the Examining Board is a political thing altogether, and the fitness of the appointees is never taken into consideration. In such cases it would be hardly fair for the board to be set off against a first-class college as a judge of the qualifications of any man to practise dentistry.

*Dr. Werner.*—If you expect to regulate the standing of the profession, you must separate it from politics. The standard of our profession should require a certain degree of skill in the technique of dental operations, a certain amount of knowledge of parts in which work is performed, and a good moral character and reputation.

In the broad, general sense, Dr. Belyea's arguments are just; but does it tend to elevate the character and tone of the profession to admit everybody who can fill a tooth? When these Examining Boards were first created it was the result of an impetus in the profession to raise the standard; to drive men into the dental schools for their education, to stop everybody filling and making teeth who had only a vague idea of what they were doing. That was the impetus that started the Examining Boards. After they were created it was found that they had an immense power, and that the persons possessing this power were not always acting for the best interests of the profession. A candidate comes before them whom they see for the first time; they never heard of him before, and do not know whether he has been in the penitentiary or has been guilty of some misdemeanor which should have sent him to State prison; yet he passes the examination and is afterwards known as a dentist and as one of our profession. Such persons may lower the dental profession in the minds of those knowing their character. Before allowing an Examining Board to have the power to grant to any man the name and dignity of belonging to our profession, I should want that board at least to come up to the standard which they want to impose upon others. I wish our profession in this matter could be regulated as it is in Germany. The board of medical or dental examiners is not appointed by a governor who is elected every year, and who, for political reasons, may make room for inefficient appointees,—no, sir; the board there is the very cream of the profession,—absolutely just and capable, something like our Supreme Court of the United States. Such men decide who shall enter the profession, and that's the way I would like to see the matter regulated here.

*Dr. Fillebrown.*—I do not think it is wise to waste too much time in discussing the side issues. The relation of the dental schools and the Examining Board is not the question to-night, neither is the question whether the Examining Boards are better than the dental faculties, but it is simply this: Shall a man be required to have passed a reasonable time in study, and to have passed a reasonable examination before his teachers and be pronounced qualified before he shall go up before the Examining Board for their approbation? Now, I, for one, believe that is where we are to come to in the future. I do not think that public opinion would sustain such a law to-day, but I think they are gradually demanding a higher standard. I do thoroughly believe it ought to be, because men will be none too well qualified even when fully up to the requirements of a graduate. I wish that we could give a unanimous vote in the affirmative on this subject,—do we think it advisable that public opinion crystallize into such a form as will require of every candidate a dental degree before he can go before a dental board for examination? I move that the answer which I have read to you be adopted.

*Dr. Smith.*—I cannot agree with Professor Fillebrown in his interpretation of this question. The question reads: "Should not the granting of certificates of qualifications by Examining Boards to non-graduates be generally abolished?" In other words, do we think or do we not think that the Examining Boards should have the power to say to non-graduates or undergraduates, when in their judgment it seems best to do so, "You must present to us a degree before we will examine you." Now, I hold that the Examining Board has nothing whatever to do with a man's position, other than that he be a man of good moral character; if so, and he makes proper application, then the Examining Board is in duty bound to examine him, and it should be so. I do not think that Examining Boards have the right, nor should they have the right to make any distinction in persons who present themselves for examination. I do think, however, that the matter should be controlled; but I hold that it can and should be controlled by the schools acting in concert. The schools could exercise an important influence in this matter if they would formulate a rule, which would be the rule of the National Association of Faculties, and every school would rigidly enforce it, that no student should present himself to the State Board for examination so long as he was a member of that school. That would settle the question. If, on the other hand, the proposition which Dr. Fillebrown makes and wishes us to vote upon was to become a law, that would necessitate the State in every instance requiring a degree of a man before he is eligible to his examination, and, as Dr. Belyea says, we can conceive of instances where this would be unfair. I do think that it has a demoralizing effect, for undergraduates to take the examination before they get their degree, for this reason: there are students at the end of their second year who know more about dentistry, in their own estimation, than they ever will again, and there is no doubt in their minds that they are fully qualified to practise. Such students go up for examination, and, if successful, immediately open an office. Then the attention is divided between practice and study, and the inability to devote a sufficient amount of time to either results is a detriment to school interests, a detriment to his own interests, a detriment to his patients' interests, and a detriment to the interests of the profession. I think that state of things should be abolished, but I do not see how the Examining Boards have the power to do it. The question, as I read it, is whether we think the Examining Board should or should not have the power to refuse the granting of certificates?

*Dr. Fillebrown.*—They cannot refuse to grant them.

*Dr. Smith.*—That's what I say. The question we are asked to express our opinion upon is not as to whether the faculties are better than the Exmining Boards, or whether the appointment of the Examining Board is a political matter.

*Dr. Fillebrown.*—It seems to me that Dr. Smith has confined his remarks to the matter of undergraduates. I do not understand that this question mentions undergraduates at all. It refers simply to any citizen of the State who is not a graduate of a dental school.

*Dr. Smith.*—An undergraduate is a non-graduate, and is therefore included in this question.

*Dr. Fillebrown.*—We are asked to express our opinion as to whether or not the granting of certificates of qualification by Examining Boards should be generally abolished. If it is abolished it must be done by State law, by legislative enactment. They have no authority to refuse to grant a certificate of qualification to any citizen of the State who passes their examination, whether he be twenty-one or forty. They have to do it. If they do not do it they lay themselves liable to the law. One of our students can go up and ask to be examined, and they have no power to refuse him. They do not ask him, by right, whether he is a student or not; and, if he passes an examination as good as the average of the men who come before them, they must give him a certificate of qualification to practise dentistry in the State of Massachusetts. I believe men should be compelled to study their profession before they are given their license to practise. I do not believe the American public is quite ready to take this position, and yet I do not believe they will be satisfied to remain long behind the place where our European friends are,—when they will demand that the examination through which a man shall pass in order to obtain his license to practise shall be severe enough to leave no doubt as to his qualification. I framed my answer, looking somewhat into the rosy future, not content with the immediate present.

*Chairman Barker.*—If I may be permitted to formulate what I consider the gist of the matter, I would put it in this way, Should the possession of a dental diploma be held to be a prerequisite to examination before a Dental Examining Board?

*Dr. Fillebrown.*—That is reframing the question, not the answer.

*Dr. Smith.*—I fully second that motion by Dr. Fillebrown. I have chosen to speak on this subject from a point of view different from his, because I felt that the question referred largely to undergraduates in schools; but, as the question is now put, that is really the only professional answer we can give to it.

*Dr. Fillebrown.*—The Examining Board has no power in this matter. You must give them this power by law. All we can do is to express our opinion as to what the law ought to be.

It was then voted to adopt Dr. Fillebrown's answer to this question.

*Dr. Fillebrown.*—Question three:

"To what extent is the washing of amalgam fillings an important feature in the production of a good filling?"

This answer, of course, embodies my own feelings and opinions, so far as I have been able to gather from my practice and association with my brethren for a long time.

"Washing of amalgam produces more perfect amalgamation and makes a finer and smoother filling; it also lessens the tendency of amalgam to shrink or change its form."

*A member.*—What do you wash it with?

*Dr. Fillebrown.*—Soap and water, or other alkali, or dilute acid.

*Dr. Taft.*—I would like to ask Dr. Fillebrown on what ground he bases his opinion that the washing of amalgam with "soap and water or anything else" will lessen its shrinkage and make it a better filling?

*Dr. Fillebrown.*—I did not feel that this was an occasion to go into the discussion of that point. This is merely an opinion of the Academy which is to be expressed by the vote of the greatest number. I believe that the washing of amalgam makes a better filling, some of you perhaps believe that it does not, and I think the quickest way is to come to a vote on it at once, for we may discuss it for an hour here and it will not alter our opinions.

*Dr. Bradley.*—I think the answer should depend somewhat upon the quality of the amalgam. Some amalgams, perhaps, need washing; with others there is no necessity for doing it. My practice is never to wash amalgams, and I think I make as good amalgam fillings as if I did wash the amalgam.

*Dr. Pond.*—I have made experiments, and yet I have only an opinion. I tested some of the fillings made in each way by putting them in a steel die and crushing them, but was unable to demonstrate any material difference. I do not wash the clean alloys. Some of the old alloys were very dirty, and those I wash; but the alloy I have recently used is clean and bright and makes a perfect filling, and I do not wash it.

*Dr. Potter.*—I would suggest that we signify in some way what we do practically in our work,—whether we wash amalgams, or whether we do not, and it will be better than to theorize as to what the effect might have been if we had or had not washed the amalgam in certain fillings. It is evidently the wish of the committee to find out whether the majority of dentists in this country wash their amalgams or not, I think we ought to send an expression of our practice rather than of our theory.

*Dr. Smith.*—I do not think that Dr. Fillebrown's answer to this question is one which the Academy should be willing to send out as its own answer without first having made a scientific investigation of this matter. It may be that he has made such an investigation, and if so, we shall be very glad to hear the results he has obtained. I doubt, however, if he will claim to have done it. Now, what do we mean by a scientific investigation of this subject? It simply means this: That you must take the different makes of amalgams, mix them and insert them without washing them, and then take those same amalgams and insert them after washing them; keep a tabulated record of all the cases, and at every opportunity note the condition of all the amalgam fillings you have put in. Now, what member has done it? The member that has not done it certainly cannot give a scientific answer to my question. Some amalgam fillings where I have washed the amalgam have not turned out as well as I had hoped for. Others where I did not wash the amalgam have turned out exactly in the same way; consequently I cannot say whether it was an improvement to wash the amalgam or not. The majority of the amalgam fillings that I have put in have not been washed, and a comparatively larger number of those seemed to me to stand better than those that I did wash. I do not claim that washing did them injury; there may have been other factors which would affect the wear of the fillings, or it may have been the amalgam itself. I do not know what the cause was, because I have never scientifically investigated it, and I do not see how the society can intelligently answer this question. I do not think we ought to vote to make an answer or statement on this subject before making careful investigation of it.

*Dr. Fillebrown.*—I want to disclaim any intention or effort to lead the society into making statements or answers which they may not fully concur in. It was necessary to open the subject in some way so as to get the expression of the society in reference to the question asked by the committee. In this particular matter I really do not feel greatly interested; I should not have raised the question myself, and I would not have formulated an answer, except to bring it before you. You may change the answer and make it read to the satisfaction of the majority.

*Dr. Eames.*—I might say that I make quite a complete record of all the circumstances attending operations. I am not prepared to say just what proportion of amalgam fillings I have washed and what the result has been, but I have an idea from an examination of such after the record has been made that I have better results from the washing than in those cases where I have not washed the amalgam. It has been proposed that we state our practice. My practice is to wash amalgam, especially if it has some age. I have been told by one of the men of the S. S. White Dental Manufacturing Company that many practitioners call for an old amalgam. That, according to my mind, is contrary to both theory and practice. I want comparatively new amalgams. I understand from what Dr. Pond has said that if he has a new or a good quality, bright amalgam, one which is not oxidized, he does not wash it; if he is obliged to use one which he considers an inferior grade, he does wash it. To sum up the matter, he believes in having a clean amalgam, and that is exactly my feeling. If you have a bright surface, the mercury will flow over it freely so that it takes less to amalgamate the alloy. I believe that the less mercury you have in a filling the better the result.

*Dr. Werner.*—I do not want to detain the Academy on this question; I think it should be passed by quickly. As Dr. Smith has said, the society cannot give a definite answer to it; you can simply pass an opinion as to what should be good practice, and let the society give as its answer an expression of the practice of the majority. Some of us cannot vote either way.

*Dr. Fillebrown.*—Would it be reasonable to note the number present at the meeting this evening, and to report as our answer that so many believe that washing amalgam improves its value as a filling, and so many do not?

*Dr. Smith.*—Many do not know whether it does or not? I think there are quite a number of us in that situation.

*Dr. Fillebrown.*—Suppose that I substitute for the answer which I first made the following:

"That the society wishes to express itself as being undetermined as to whether amalgams are improved to any extent by washing or not."

Answer adopted.

Just one question more, now:

"What results are to be expected in replantation or transplantation as a means of treatment of chronic phagedenic pericementitis?"

I will say,—

"No marked results for good can be expected from replantation; the transplantation of healthy teeth into a diseased jaw will improve the condition of the relations of the tooth and the jaw."

I mean by the latter phrase that you will have a better union and a firmer tooth in the socket than the one that was taken out.

*Dr. Smith.*—It seems to me that this again is a question that cannot be voted upon, yes and no, by the Academy, and the vote of the majority sent to be recorded as the society's answer to that question. This is a question that must be answered by experience. What members have had the experience with this method of treating this condition of affairs? Personally, I have never had any experience whatever. I have never replanted a tooth, and I have never transplanted a tooth; therefore, I cannot vote either way.

It occurred to me that what was intended by the committee in sending out this question was to get the experience of as many as possible with this method of treatment; that experience is to be formulated; then the collector of such literature in the future goes over those statements, and a method of treatment is laid out, which, based upon experience, is declared to be the best that can be done under certain conditions. It seems to be quite wrong to put the matter in the form of a vote by the entire Academy when some of us know nothing about it.

*Dr. Fillebrown.*—I think the point raised by Dr. Smith is well taken. I will simply then give the answer which I have just read as my opinion, based on my own experience, of course, in a very few cases,—that is, that replantation is no good whatever; that transplantation of a healthy tooth does improve the condition.

*Dr. Stevens.*—How many gentlemen in the room have ever replanted a tooth which had Riggs's disease?

Drs. Fillebrown, Eames, and Draper replied that they had. Dr. Preston had extracted several teeth, filled and replaced them, and the operations were successful. In the case of an abscessed tooth in which he tried it, it was not successful. He had never attempted it where there was disease of the socket.

*Dr. Eames.*—I have tried replantation in a couple of instances, but they were desperate cases. I hardly think any one would resort to that until the tooth was practically lost, and that was the condition in both of my cases. They were both in the lower jaw. I replanted, after the thorough removal of any deposit on the tooth, cleansing and repairing the socket, and I must say there was an improvement. The teeth in both cases were considerably strengthened in their positions, were firmer with considerably less soreness, but the teeth were not serviceable, and were finally extracted.

*Dr. Smith.*—I would like to ask why this question was put in this form? You may have a case of Riggs's disease without having chronic phagedenic pericementitis. The idea of "phagedenic" as given by Gould is that of a "rapidly spreading and destructive ulceration." We find many cases of Riggs's disease where we do not find that condition.

*Dr. Daly.*—I understood this question in the same way,—that is, that it referred to an ulcer.

*Dr. Fillebrown.*—So did I. "Phagedenic" means ulcerative.

*Dr. Eames.*—I will simply say that cases I referred to were not phagedenic pericementitis, as I understand that term.

*Dr. Fillebrown.*—Dr. Black says that phagedenic pericementitis includes Riggs's disease, pericementitis. That is Dr. Black's position, and it has always appeared to me as being a reasonable one.

Subject passed.

*Dr. Clapp.*—I have a large number of patients, elderly people, who had originally very good teeth. As they advanced in age there was marked recession of the gums, absorption of the process, leaving more or less of the roots of the teeth exposed. As age still advanced, there seemed to be a sort of decadence of the substance of these roots,—I suppose it would be termed scientifically a sort of a "retrograde metamorphosis." As these roots decayed, I found them very difficult to treat satisfactorily. I have used in the anterior teeth the various cements and gutta-perchas, with poor results. In few cases I have tried gold combined with cement,—crowding the first pieces of gold into soft cement that was placed in the cavity and finishing with gold after the cement had hardened. This is not a very satisfactory method for these teeth, because in many cases the teeth are loose and the operation is extensive and too fatiguing for the patient. For the posterior teeth I have used with the best satisfaction amalgam which I have placed in the cavities in combination with soft cement. As I said before, it is not to tell what I have done, excepting in matter of failures, but to receive some knowledge that I wished to bring this question before you.

*Dr. Daly.*—The best man to answer that question is Dr. Adams.

*Dr. Adams.*—I think the gold bandage is admirably adapted for such cases,—teeth where the necks are exposed and the surfaces softened. I scrape off the softened surface of the tooth, wipe it over with a strong solution of nitrate of silver, fill the cavities if there are any, restoring the contour with oxyphosphate, and apply the bandage; and, so far as my experience has gone, it has worked very satisfactorily. Some of you, perhaps, have not heard of this operation, and if any one who has not thinks he would be interested in it, he can find the process described in the January number of the *Dental Digest.*

*Dr. Clapp.*—How long does it take to treat an ordinary case of this kind?

*Dr. Adams.*—I should think perhaps two hours. The bandage covers the whole surface, and I think the chances of any future decay are small.

*Dr. Stevens.*—Will not Dr. Adams give a description of his method for the benefit of those who have not read his article?

*Dr. Adams.*—The process is quite fully described in the article referred to, and it is probably a better explanation of it than I can give you here. I simply cut a slot in that part of the tooth which I wish to cover (this only applies to anterior teeth), and, winding the bandage around tightly, place the ends into that slot and fill the slot with gold. The bandage is made of thirty-four-gauge pure gold and lined with cement.

*Dr. Stevens.*—It seems to me that any one who has tried Dr. Adams's method would say at once that there can be no improvement on it.

*Dr. Smith.*—Do I understand you to say that it does not well apply to molars?

*Dr. Adams.*—I have never tried it on molars. Perhaps in some cases you could get at the first molars so as to treat them in that way.

*Dr. Smith.*—The most difficult cases we have are those where decay has commenced at the necks of molar teeth, extending far under the gum and following down the roots of the tooth. It is very difficult to spread such teeth and secure sufficient room; and to fill them with something that is at all lasting is an exceedingly difficult task, because, even if you succeed in clearing out the decay, it is almost impossible to keep cavities dry. If it was a cavity that communicated with the crown, you might get at it by continuing the excavation from the crown cavity, first applying the matrix; but in the cases I have just referred to you cannot use the matrix, and their excavation and treatment is a source of great discomfort to the operator and to the patient. For a long time I filled such cases with gutta-percha and cement, until I became thoroughly tired of repairing and replenishing; and I then adopted the use of amalgam, and I have been a great deal better satisfied with the results, although it is by no means as satisfactory as we would like. In some cases the patient will use a toothpick rather vigorously and the gutta-percha or cement filling, which cost us so much trouble to put in, is out in an instant; and then, in some mouths the acid secretions quickly wear out the cements, so that we always feel uncertain in the use of cements in such places. Of course, those gentlemen who do not use amalgam—nothing but gold—are not troubled with this uncertainty, but just how they succeed in placing in gold in the class of cases we are referring to is beyond my understanding. I think those are cases where a man had better cast aside prejudice and use a little amalgam, providing his patients will allow it; if not, I see no other course than to put in cement and gutta-percha and resignedly accept the task of replenishing. I think it would be very interesting, Mr. President, for those gentlemen who do not use amalgam to state how they treat such cases. Some one may have a method of treating those places; if he would only tell it, that would be of great benefit to the rest of us. At all events, I should like to hear a full discussion of these troublesome places and how best to manage them. It is a very important subject, it seems to me.

*Dr. Williams.*—I look upon the class of cases under discussion much as a physician would a chronic disease. He does not expect to give one dose of medicine with the expectation that it will cure the thing permanently, but that it will require a course of treatment. The course of treatment that I have followed in these cases is to fill them with an amalgam, but I make a special amalgam of pure tin. I take a piece of block tin and file it, and these filings combined with the mercury make a very soft amalgam. The object is to get an oxidation of the tin, and with a slight moisture of the cavity there surely will be an oxidation of the tin. My method is to wipe the cavity, after careful excavation, with dry oxide of tin, put in my soft amalgam, and then rub over the surface of it some regular amalgam, which gives it a better wearing surface. I do not intend, however, to allow that filling to remain permanently; I call it a treatment, and look upon it just as you would a treatment with gutta-percha or cement which is to be kept under observation for several months. At the end of that time, almost invariably, there is a greater or less degree of hardening of the dentine, then you may get a retaining point which you would reasonably expect to hold a more permanent filling. Many operators hope finally to get this result by using cement, but the amalgam treatment seems to me to have the most direct effect in hardening the dentine in these cases,—amalgam with a good proportion of tin in it.

There is one possibility in connection with this method,—you must remember that it is very soft amalgam, and if some other dentist happens to see the filling, he would be very likely to want to know who put it in, and say "anybody ought to know enough not to use such soft stuff as that," not understanding that it was intended as a dressing as a treatment preparatory to a more permanent filling.

*Dr. Stevens.*—One word in regard to putting amalgam into the faces of front teeth. I do not think I could be persuaded to do it even at the emphatic request of the patient. I do not see any necessity for it when you can make porcelain inlays to fit any place of that kind which you wish to repair, and if I could not do that, I think I should prefer to cut the teeth off and put on crowns rather than have an unsightly amalgam filling exposed in the front of the mouth. I do not understand why you should have any difficulty in putting in a porcelain inlay, or why you should expect it to wash out. The cavity may be any shape imaginable, but if you bake an inlay for it, it will fit.

Subject passed.

*Chairman Barker.*—We will now listen to Dr. H. A. Baker, whose subject is, "Completed Regulating Case. Photographs and models. Presented as incomplete last year."

*Dr. Baker.*—I will commence with this case where we left off last year. I presented to you the photographs and models of this young man before anything was done. I also described to you a plan which I had mapped out for bringing the teeth into line. The lower jaw was receding and the upper jaw was protruding to the extent of something more than half an inch, so that the lower incisors hit the gum on the upper jaw nearly an inch back. I used here the Patrick system altogether. I have here the bands with which I spread the jaws and brought the upper jaw back and the lower jaw out. I waited for this case until it was ripe,—just before the eruption of the twelfth year molar, thinking it would assist in the regulation of the teeth. I put the upper band on June 29 last; the lower band I put on July 1. I then tied each tooth front of the molars to the band, and screwed the nuts up, and that spread the jaw both ways. You will see in the corrected model that I have jumped the bite about one tooth. I have seen many cases of umping the bite, but I never saw a case where it was more perfectly done than in this one. The upper jaw was spread about one-quarter of an inch and the lower one to the same extent. This young man has a receding chin. I do not know of any way to bring a person's chin out, or I might have attempted it and made one job out of the whole thing. August 29, about two months from the commencement of the work, I took off the anchor band and simply put on two wires, tying them to the teeth. The case was practically completed the 29th day of August.

I want to claim the originality in the matter of this retaining plate. We all know that to retain teeth is much harder than to regulate them, and to get a plate that will not produce decay is one of the difficult things that I have had to contend with in the regulation of teeth. In most of the apparatus for retaining teeth they begin in the first place by capping the teeth all over. In this upper case I simply made bands attached to the molars with a projection on the palatal surface, so that the wire from this plate snaps under there. In the lower case you will observe that the plate has come back, or the jaw has come forward (more likely the former) about one-sixteenth of an inch. In this case I made a spur running from the plate against the molar, between the molar and the first bicuspid, to prevent its going back, and it does its work beautifully.

William H. Potter, D.M.D.,
*Editor American Academy Dental Science.*

